# Proinflammatory Effects of C-Peptide in Different Tissues

**DOI:** 10.1155/2012/932725

**Published:** 2012-06-11

**Authors:** Dusica Vasic, Daniel Walcher

**Affiliations:** Department of Internal Medicine II, Cardiology, University of Ulm, Albert-Einstein-Alle 23, 89081 Ulm, Germany

## Abstract

Atherosclerosis is well known as an inflammatory disease that can lead to clinical complications such as heart attack or stroke. C-peptide as a cleavage product of proinsulin is in the last few decades known as an active peptide with a number of different effects on microvascular and macrovascular complications in type 2 diabetic patients. Patients with insulin resistance and early type 2 diabetes show elevated levels of C-peptide in blood. Several last findings demonstrated deposition of C-peptide in the vessel wall in ApoE-deficient mice and induction of local inflammation. Besides that, C-peptide has proliferative effects on human mesangial cells. This review discusses recently published proinflammatory effects of C-peptide in different tissues.

## 1. Structure of C-Peptide 

C-peptide is a small peptide of 31 amino acids and short half-life of approximately 30 minutes. It has been identified by Steiner 1967 as a by-product of proinsulin and its main role was in assisting in the arrangement of the correct structure of insulin [1]. Proinsulin consists of an A chain, connecting peptide (C-peptide), and B chain. C-peptide has a central glycine-rich region which allows a correct positioning of A and B chains for insulin to achieve its tertiary structure [1]. It is secreted into the bloodstream in equimolar amounts together with insulin in response to glucose stimulation. C-peptide has been since a long time considered as an inactive peptide. However, over the last two decades, numerous studies revealed that C-peptide displays a physiological role in different cell types [2, 3]. C-terminal pentapeptide of C-peptide obtains the full activity of intact C-peptide in stimulating Na^+^/K^+^-ATPase [4]. Amino acid sequence of C-peptide is in different species relatively variable, although it has several conserved sequence like N-terminal acidic region, glycine-rich central segment, and C-terminal pentapeptide [5]. Binding of C-peptide was investigated by fluorescence correlation spectroscopy. The authors find C-peptide binding to the cell membranes of intact fibroblasts with the saturation at the physiological levels of C-peptide [6]. Although C-peptide receptor remains unknown, it has already been shown that C-peptide activates signaling pathways in different cell types. For example, it binds to pertussis-toxin-sensitive G-protein-coupled receptor on Swiss 3T3 fibroblasts [7] and activates p38 protein kinase pathway in mouse lung capillary endothelial cells [8, 9]. Effects of C-peptide have a positive influence on long-term complications in type 1 diabetic patients. C-peptide has an impact on diabetic neuropathy via improvements of endoneural blood flow and axonal swelling [10] or improves decreased blood flow in extremities. [11]. Several studies proposed direct role of endogenous insulin and C-peptide in improvement of endothelial dysfunction [12]. Moreover, C-peptide increases nitric oxide (NO) production through ERK1/2 MAP kinase-dependent up-regulation of endothelial nitric oxide synthase (eNOS) gene transcription [13].

The effects of C-peptide in type 2 diabetes and cell proliferation are controversial. The metabolic syndrome, prediabetes, and type 2 diabetes mellitus accelerate vascular disease and increase development of the disease [14].

## 2. Proinflammatory Effects of C-Peptide in the Vasculature

First reports about the C-peptide deposition in the vessel wall came from Marx et al., when they demonstrated deposition of C-peptide in the subendothelial space in thoracic aorta in diabetic subjects [15]. In this study, it was found the C-peptide deposition in intima of the vessel wall in the thoracic aorta of diabetic subjects. From 21 subjects with deposition of C-peptide, 77% showed infiltration of monocytes/macrophages and 57% infiltration of CD4^+^ lymphocytes [15]. In further studies, *in vitro* migration assays reported that C-peptide induces migration of CD4^+^ lymphocytes and monocytes/macrophages in a concentration-dependent manner. These effects were similar to those induced by monocyte chemokine MCP-1 or T-lymphocyte chemokine RANTES. Checkerboard analysis in the same study shows that C-peptide induces chemotaxis rather than chemokinesis with maximal effect that correspond to physiological concentrations of C-peptide (1 nmol/L) [15, 16]. C-peptide mediates its chemotactic activity in CD4^+^ lymphocytes and in monocytes through an as of yet unidentified pertussis toxin-sensitive G-protein coupled receptor and stimulates specific intracellular signaling pathways in these cells [17]. C-peptide stimulates similar signaling pathways in different cell types. For example, Na^+^/K^+^ATPase [4, 18], ERK1/2 MAP kinase, and PI-3 kinase [9, 16, 19, 20]. Aleksic et al. revealed that activation of PI-3 kinase*γ* induced by supraphysiological concentrations (10 nmol/L) of C-peptide leads to an activation of Rho GTPases. Rho, Rac1, and Cdc42 are small GTP-binding proteins with GTPase activity. Activation of Src-kinase and RhoA, Rac-1, and Cdc42 GTPases act via PAKs (p21 activated kinase) and stimulate LIMK (LIM domain-containing protein kinase), which phosphorylates and inhibits cofilin. This leads to increased accumulation of polymerized actin at the leading edge of cells. RhoA stimulates MLC (myosin light chain) phosphorylation via ROCK (Rho kinase) activation which is important for cell body contraction and migration [17]. C-peptide positively controls the expression of the PPAR*γ*-regulated CD36 scavenger receptor in human THP-1 monocytes. Its stimulates PPAR*γ* activity in a ligand-independent fashion and this effect is mediated by PI-3 kinase [21]. 

Further, effects of C-peptide on smooth muscle cell proliferation have been investigated. Walcher et al. showed that high levels (10 nmol/L) of C-peptide induces proliferation of human and rat smooth muscle cells in concentration-dependent manner assessed by Ki-67 assay and 3[H] Thymidin assay. Extent of proliferation was similar to those induced by platelet-derived growth factor (PDGF) [19]. In addition, C-peptide induces phosphorylation of protein tyrosine kinase (Src) and PI-3 kinase. Further, it induces activation of specific ERK1/2 MAP kinase [19]. VSMC proliferation by extracellular stimuli takes place in mid-to-late G_1_ phase of the cell cycle, where D-type cyclins promote G_1_- to S-phase transition by leading to Rb phosphorylation [22]. Walcher et al. showed that C-peptide increases cyclin D1 expression and Rb phosphorylation that suggests that C-peptide acts via similar signaling pathways [19]. In another study, Insulin cannot alter endothelial cell (EC) proliferation or migration, where 10 nmol/L C-peptide stimulates EC proliferation by 40% [23]. Proliferation effects of C-peptide have been shown in different cell types, for example, like endothelial cells, HEK293 cells, and chondrocytes. Lindhal et al. found that C-peptide stimulates rRNA synthesis and induces expression of 47S in HCS-2/8 chondrocytes derived from a human chondrosarcoma. This regulation of ribosomal RNA provides amechanism by which C-peptide can apply its transcriptional effects and its growth factor activity [24].

**Figure 1:**
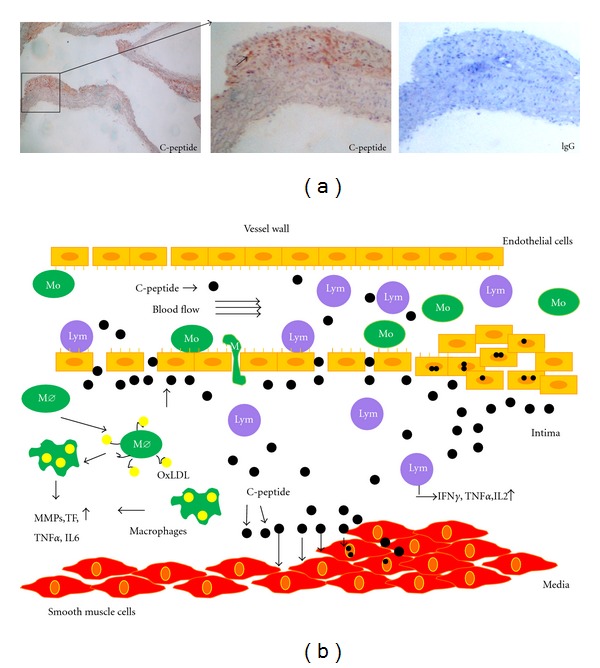
C-peptide deposits in mouse aortic arch. Red areas represent C-peptide deposition indicated by arrow on the high power view. Adjacent sections represent negative control and show no immunoreactive C-peptide areas (Figure 1(a)). Lower panel (Figure 1(b)) illustrates potential hypothesis about C-peptide effects in vessel wall. Patients with insulin resistance and type 2 diabetes show increased levels of C-peptide in the blood. Together with endothelial dysfunction and increased endothelial permeability C-peptide deposits in the intima of the vessel wall and from there induces recruitment of inflammatory cells and their migration into the subendothelial layer. C-peptide deposits also in the media and has an effect on the proliferation of smooth muscle cells.

Summarizing these results our group tested initial hypothesis show on in the Figure 1(b). Patients with early diabetes type 2 and insulin resistance show increased levels of C-peptide in blood. Together with increased endothelial dysfunction, this leads to deposition of C-peptide in the intima of the vessel wall. According to the *in vitro* results, C-peptide may have chemotactic effect on the inflammatory cells involved in the onset of the atherosclerosis, like monocytes/macrophages and CD4^+^ lymphocytes. Further, C-peptide has an effect on the proliferation of smooth muscle cells in the media. These cells migrate into developing atheroma and together with inflammatory cell recruitment represent initial step in the developing of atherosclerosis. 

To test the hypothesis in an animal model, we used ApoE deficient mice. The animals were divided into two groups. C-peptide group numbered 18, and placebo 17 mice per group [25]. Subcutaneous injections (200 nmol/injection) of dissolved peptide increased blood C-peptide levels 5 to 6 folds compared to basal levels (12.9 ± 1.8 nmol/L compared with 2.7 ± 0.8  nmol/L; C-peptide versus placebo; *P* < 0.05). At the same time, mice were put on the Western type diet to trigger atherosclerosis. C-peptide deposition was found in the vessel wall of aortic arch and in early atherosclerotic lesions (Figure 1(a)). Computer-assisted image quantification revealed significantly higher deposition of C-peptide in treated mice, compared to placebo one (2.1 ± 0.4 versus 0.8 ± 0.1%  positive area; *P* < 0.01) treated with water. Similar results were obtained in the aortic root (data not shown). This deposition of C-peptide was followed with increased local inflammation in aortic arch. After immunohistochemical staining, computer-assisted image quantification showed increased infiltration of monocytes/macrophages in the vessel wall. Further, we know that diabetes accelerates smooth muscle cell proliferation in atherosclerotic lesions and that it correlates with insulin levels [26]. Smooth muscle cells and their secreted products are the main components of advanced atherosclerotic lesions [27]. Staining of aortic arch in ApoE–deficient mice for *α*-actin showed increased content of smooth muscle cells in C-peptide-treated group (C-peptide versus placebo; 4.8 ± 0.6 versus 2.4 ± 0.7% positive area; *P* < 0.01) as well as a trend towards more Ki-67 proliferated cells in C-peptide treated group [25]. Analysis of lipid deposition in placebo and C-peptide treated mice revealed increased deposition of lipids stained with Oil-red-O in C-peptide-treated mice compared to placebo. Lipid deposition in *en face* preparations of the abdominal and thoracic aorta in C-peptide-treated mice did not reach statistical significance compared to placebo mice (C-peptide versus placebo; 5.64 ± 0.69% versus 3.98 ± 0.5%  *P* = 0.07) [25]. Proinflammatory effects of C-peptide were obtained in the ApoE-deficient animals on top of a high cholesterol diet. Effects of high cholesterol diet can partly cover the proinflammatory effects of C-peptide in this model.

Our study revealed no differences in E-selectin and ICAM-1 levels as well as levels of the inflammatory markers such as TNF*α* and soluble IL-6, that is, in contrast to several findings where C-peptide has anti-inflammatory effects and reduces upregulation of cell adhesion molecules under inflammatory conditions [28, 29]. C-peptide is nowadays recognized as an active peptide with various effects. Further work is needed to identify C-peptide receptor and elucidate mechanisms by which it modulates cell signaling in different cell types. Different effects in type 1 and 2 diabetes seem to be tissue and cell specific. 

## 3. Proinflammatory Effects of C-peptide in Kidneys

We already know that C-peptide administration in replacement dose given to diabetic rats limits or prevents glomerular hypertrophy and mesangial matrix expression [30]. In several further studies, C-peptide reduces glomerular hyperfiltration, hypertrophy, and proteinuria [31–33]. Lower C-peptide levels are connected with increased albuminuria, retinopathy, and nephropathy [34] whereas other studies did not show relation between C-peptide levels and microangiopathic diabetic complications [20, 35]. In our previous work, we demonstrate that C-peptide exhibits mitogenic activity on human mesangial cells (MCs). High levels of C-peptide (10 nmol/L) induce proliferation of kidney human mesangial cells in a concentration-dependent manner assessed by Ki-67 assay with maximal induction of 2.6 ± 0.4 folds. Further, pretreatment of cells with inhibitors PP2 (Src kinase inhibitor) or PD98059 (MEK 1 inhibitor) decreases C-peptide-induced human mesangial cell (MC) proliferation. As well, pretreatment of cells with PI-3 kinase inhibitor wortmannin also reduces human MC proliferation. These results suggest the involvement of Src-kinase, ERK1/2 MAP kinase, and PI-3 kinase as downstream elements of the signaling pathway. We further investigated the activation of signaling pathways involved in C-peptide-induced proliferation of mesangial cells. C-peptide activates phosphorylation of Src that leads to activation of PI-3 kinase and involvement of ERK1/2 MAP kinase. High-concentration C-peptide (10 nmol/L) increases phosphorylation of ERK1/2 MAP kinase in human MCs in a time-dependent manner with a maximal effect after 10 minutes. Cyclin D activates cyclin-dependent kinase 4 (CDK4) during G1 phase that leads to phosphorylation of retinoblastoma tumor suppressor protein (pRb) [36]. C-peptide stimulation increases activation of cyclin D1 and phosphorylation of Rb suggesting that C-peptide-induced proliferation may use similar signaling pathways. These results are in agreement with *in vitro* data of swiss 3T3 fibroblasts, where C-peptide has been shown to activate PI-3 kinase [7]. Serum levels of C-peptide are associated with the metabolic syndrome in patients with type 2 diabetes and in diabetic patients with nephropathy and vascular disease [37]. C-peptide is eliminated from the body by kidneys [38]. In the period of insulin resistance and early type 2 diabetes increased levels of C-peptide are circulating through glomeruli and could deposit in juxtaglomerular apparatus and from there could demonstrate its mitogenetic effect on mesangial cells.

The ApoE-deficient mouse model is a conventional model for investigating atherosclerosis. These mice have greatly increased plasma lipid levels [39]. Appearance of atherosclerosis is similar to those in humans induced by ApoE deficiency called type III hyperlipoproteinemia [40]. It is known that ApoE-deficient mice with increased hyperlipidemia demonstrates glomerular injury characterized by glomerular endothelial cell activation and macrophage recruitment [41]. Elevated levels of albumin in urine serve as clinical predictors of diabetic nephropathy [42]. Apolipoprotein E modulates human mesangial cell proliferation depending on the length of stimulation and cell conditions [43]. It has been shown that mice with increased hyperlipidemia in plasma have an increased progression of renal disease [44].

**Figure 2:**
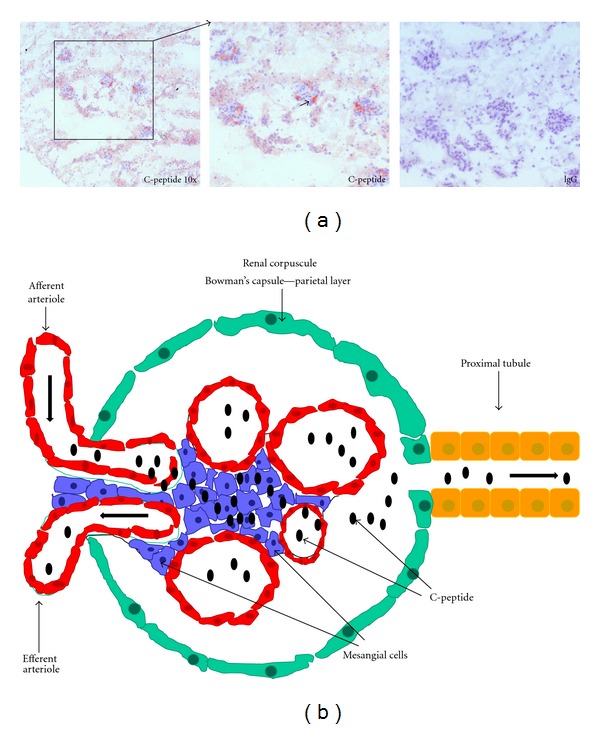
C-peptide deposition in mouse juxtaglomerular apparatus. Upper panel: Representative photograph of mouse kidney sections with C-peptide deposition in the glomeruli in C-peptide treated mice (Figure 2(a)). Red areas represented C-peptide deposition indicated by arrow. On the lower panel is schematic explained the way C-peptide induces proliferation of mesangial cells suggesting a possible role of C-peptide in glomerular hyperproliferation in patients with diabetic nephropathy (Figure 2(b)).

Assuming *in vitro* effects of C-peptide on human MCs, we further investigated deposition of C-peptide in mouse juxtaglomerular apparatus. Longitudinal sections of mouse kidneys were stained for C-peptide. Red areas in mouse glomeruli demonstrate C-peptide deposition (Figure 2). Quantitative analysis of C-peptide deposition in mouse glomeruli of ApoE-deficient mice determined increased deposition of C-peptide in glomeruli in C-peptide treated mice compared with placebo (unpublished data). Our previous work illustrated that C-peptide induces proliferation of mesangial cells, deposition in the intima and media of the vessel wall in diabetic patients, and C-peptide-induced proinflammatory effects in vascular cells. This resulted in increased C-peptide deposition in juxtaglomerular apparatus in C-peptide-treated mice. Still, the relevance of these results to human atherosclerosis or diabetic nephropathy remains to be determined.

## 4. Conclusion

In this review, we explained several proinflammatory effects of C-peptide on the inflammatory cells in the vessel wall and its mitogenic effects on the smooth muscle cells. Based on the previous results, we demonstrated that C-peptide deposits in the vessel wall in ApoE-deficient mice and induces local inflammation that leads to increased lipid deposition in aortic arch and increased proliferation of smooth muscle cells, crucial processes in the onset of atherosclerosis. Further, we explained an effect of C-peptide on the mesangial cell proliferation that involves Src kinase, PI-3 kinase, and ERK1/2 MAP kinase, and for the first time the deposition of C-peptide in mouse kidney juxtaglomerular apparatus. These results raise the hypothesis that C-peptide may have a role in glomerular hyperproliferation in patients with diabetic nephropathy.
